# Novel Tri-Specific Immuno-Modulatory Antibody Combined with HDACi to Potentiate T Cell Activation and Anti-Tumor Efficacy

**DOI:** 10.7150/jca.108922

**Published:** 2025-08-30

**Authors:** Yidan Gou, Xiangfei Yuan, Dongmei Fan, Yuanyuan Yang, Hengjie Yuan

**Affiliations:** 1 Tianjin Medical University General Hospital, Tianjin Medical University, Tianjin, China, 300052.; 2 Tianjin Key Laboratory of Acute Abdomen Disease Associated Organ Injury and ITCWM Repair, Tianjin Medical University NanKai Hospital, Tianjin, China.; 3 Tianjin Key Laboratory of Blood Disease Cell Therapy, State Key Laboratory of Experimental Hematology, National Clinical Research Center for Blood Diseases, Institute of Hematology and Blood Diseases Hospital, Chinese Academy of Medical Sciences, and Peking Union Medical College, China.

**Keywords:** Chidamide, tri‑specific antibody, PD-L1, Immunotherapy, bispecific T-cell engager

## Abstract

**Background:** Although blinatumomab, a bispecific T-cell engager, has exhibited promising clinical outcomes in the treatment of B-precursor acute lymphoblastic leukemia (B-ALL) and B cell non-Hodgkin's lymphoma (B-NHL), it still has some limitations due to CD19-negative relapse and an immunosuppressive phenotype. Epigenetic modulators have the potential to influence the tumor microenvironment (TME) and immune cycle in various ways. Combining multiple drugs is a trend in tumor treatment.

**Methods:** In this article, we generated a novel tri‑specific antibody, called CiTE (CD3-BAFF-R-PD-L1), which is composed of a CD3 binding Fab frame, a BAFF-R binding scFv, and an additional scFv derived from PD‑L1. The cytotoxicity of T cells induced by CiTE (CD3-BAFF-R-PD-L1) was detected in combination with Chidamide.

**Results:** The purified CiTE (CD3-BAFF-R-PD-L1) exhibited high binding capability for CD3-positive cells, BAFF-R-positive cells, and PD-L1-positive cells. Consequently, specific lysis towards BAFF-R-positive cell lines were induced by CiTE (CD3-BAFF-R-PD-L1) in the presence of T cells. We also confirmed that Chidamide could enhance T-cell mediated lysis by upregulating antigen strength on tumor cells and altering the TME by increasing the expression of T cell-attracting chemokines and costimulatory molecules.

**Conclusions:** The novel tri-specific antibody combined with Chidamide holds promise as a safe and effective drug for the treatment of B-cell malignancies, due to its integration of three sections targeting different molecules into one compound.

## Introduction

T cells are the most promising killer cells, and several bispecific T-cell engagers (BiTEs) have achieved significant success in treating blood cancers, providing rapid and durable responses 1. Blinatumomab, a bispecific antibody targeting CD19 and CD3, has been approved by the FDA for treating relapsed or refractory B-ALL 2. Despite its strong tumor-killing activity, many patients suffer relapses following CD19-targeted therapies and other target-dependent immunotherapeutic approaches 3, 4. The main reasons for relapse are either the loss of specific antigens on tumor cells or the exhaustion of killer cells. There is a need to find new alternative therapeutic targets for resistant B-cell malignancies. BAFF-R, expressed exclusively on B cells 5, is a particularly interesting alternative target in patients with CD19-negative relapse. BAFF-R is closely associated with the function and survival of B-cell lymphoma 6, 7, which may limit the immune escape of tumors through the downregulation of BAFF-R expression. A BAFF-R targeting CAR T cell has been constructed, demonstrating antitumor effects against a panel of human lymphoma lines and patient-derived xenografts (PDXs) *in vivo* 8.

In addition to antigen loss, the activation of immunosuppressive pathways also contributes to resistance to T-cell therapy. Enhanced expression of programmed cell death 1 (PD-1) and programmed cell death 1 ligand 1 (PD-L1) was observed upon BiTE treatment, while combined blockade of the PD-1/PD-L1 axis may be a promising strategy to promote the antitumor effect of BiTE 9, 10. In preliminary clinical trials, the combination of the PD-1 antibody cemiplimab and the CD20 BiTE REGN1979 showed acceptable safety 11.

Histone deacetylase inhibitor (HDACi), as epigenetic modulator, has been proven to induce tumor apoptosis, inhibit the cell cycle, and promote cell differentiation 12. Chidamid, an oral HDAC inhibitor of the benzamide class with specificity for HDAC1, HDAC2, HDAC3, and HDAC10 subtypes, has been approved for the treatment of relapsed or refractory peripheral T cell lymphoma. Several recent studies show that epigenetic modifiers can play critical roles in regulating both tumor cell-intrinsic immunity and T-cell exhaustion 13. Immune checkpoint inhibitors (ICI) often transiently reprogram or activate T cells to restore their effector cell-like functional phenotypes. Several epigenetic regulators suppress the expression of endogenous antigens, and the loss of these regulators results in viral mimicry reactions that enhance the response of "cold" tumors to ICIs 14, 15. Targeting the intersection of epigenetics and immune checkpoint treatment holds enormous promise to increase anticancer immune responses and herald the next generation of persistent immuno-oncology therapies 16.

**Figure 1:**
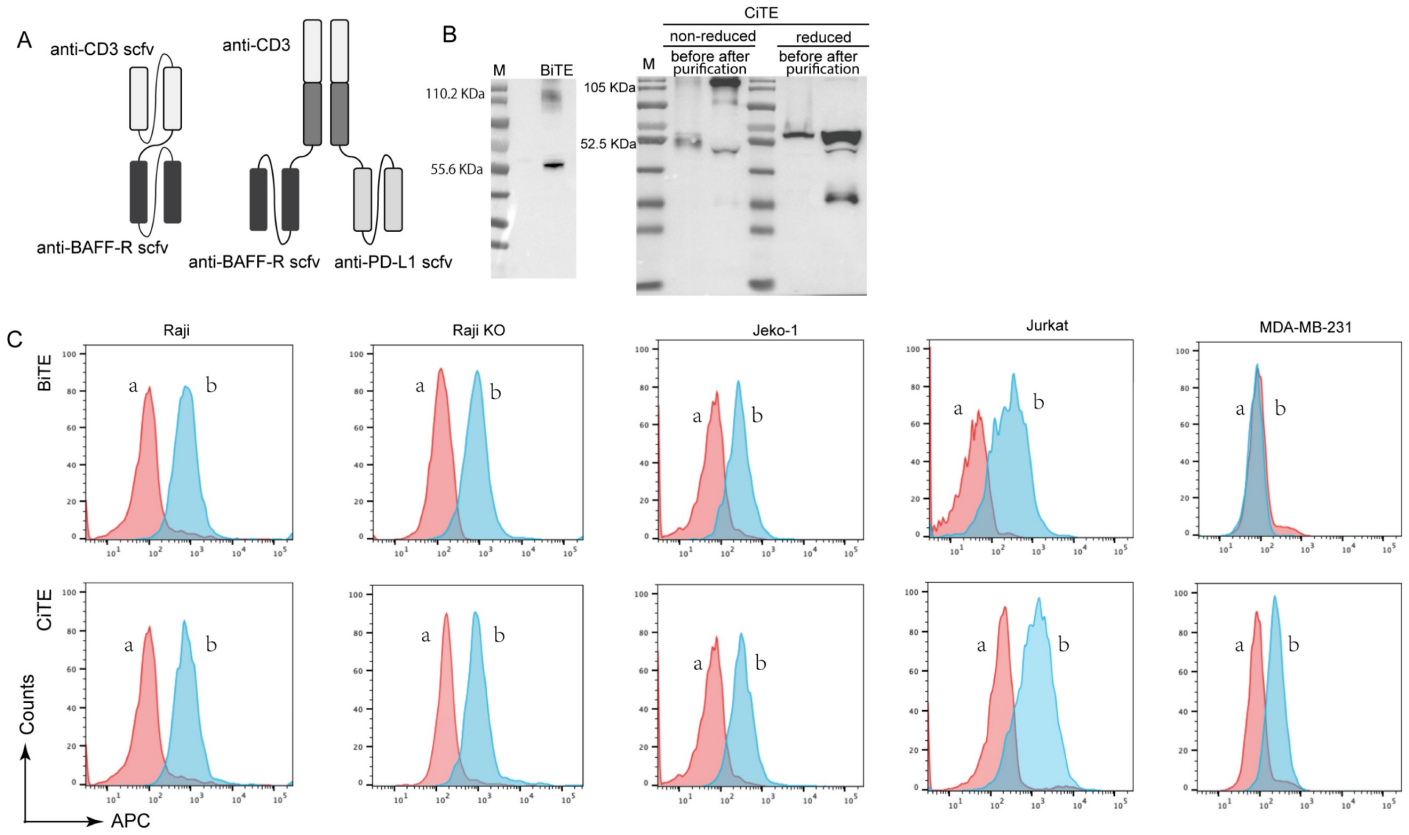
** Production and binding effect of CiTE (CD3-BAFF-R-PD-L1). (A)** Scheme of assembled tribody proteins. **(B)** BiTE (CD3-BAFF-R) and CiTE (CD3-BAFF-R-PD-L1) was detected by Western blot analysis. The supernatants from 293T cells after transfection with pcDNA3.1(+) expression vectors were harvested and assayed with anti-His tag antibodies. **(C)** Specific binding of CiTE (CD3-BAFF-R-PD-L1) to tumor cell lines. FACS analysis with the CiTE (CD3-BAFF-R-PD-L1) on CD19-positive Raji cells, CD19-KO Raji cells, Jeko-1 cells, on CD3-positive Jurkat cells, and on the PD-L1-positive MDA-MB-231 cells. a, negative controls with the secondary antibody anti-His-Alexa Fluor 488 alone; b, BiTE (CD3-BAFF-R) or CiTE (CD3-BAFF-R-PD-L1).

In this study, we constructed a tri-specific fusion protein named CiTE (CD3-BAFF-R-PD-L1), which is constituted by anti-CD3 scfv, anti-BAFF-R scfv and anti-PD-L1 scfv (Fig. 1A). We hypothesized that the tri-functional fusion protein CD3-BAFF-R-PD-L1 would be an effective means of neutralizing PD-L1-mediated immune suppression while also enhancing insufficient T cell priming, bypassing MHC recognition and impaired antigen-specific responses 17, 18. Furthermore, we analyzed the antitumor efficacy of CiTE (CD3-BAFF-R-PD-L1) in combination with HDAC inhibitor Chidamide to explore its underlying mechanisms. This work provides an approach for clinical antitumor therapy based on the combination of an epigenetic modulator and T-cell based antibody therapy.

## Materials and Methods

### Cell lines and cell culture

Human breast cancer cell line (MDA-MB-231) and human embryonic kidney cell-derived 293T cell line was maintained in DMEM (Gibco, 1791922) supplemented with 10% FBS. The human acute T cell leukemia cell line Jurkat, human B cell lymphoma cell lines Raji, Z138, Jeko-1, and SU-DHL-10 were grown in RPMI-1640 medium (Invitrogen, USA) supplemented with 2 mM L-glutamine, 100 U/mL penicillin (Gibco, USA), 100 μg/mL streptomycin (Gibco, USA), and 10% FBS. The cells were incubated at 37 °C in a humidified atmosphere containing 5% CO_2_.

### Expression and purification of BiTE (CD3-BAFF-R) and CiTE (CD3-BAFF-R-PD-L1)

293T cells were transfected with pcDNA3.1(+)-CD3-BAFF-R or pcDNA3.1(+)-CD3-BAFF-R-PD-L1 using Lipofectamine 3000 (Invitrogen, USA) according to the manufacturer's protocol. After 48 h of transfection, supernatants were collected by centrifugation at 2000 rpm for 10 min at 4 °C to clear cellular debris. The secretory BiTE (CD3-BAFF-R) and CiTE (CD3-BAFF-R-PD-L1) in the supernatants were purified by 6×His-tag affinity chromatography (GE Healthcare, Sweden), followed by buffer exchange. The purified preparations were quantified by Western blot analysis and used for follow-up activity studies.

### Binding Assays

To confirm the binding specificity of the novel generated BiTE (CD3-BAFF-R) and CiTE (CD3-BAFF-R-PD-L1), the CD19-positive cell lines Raji, the CD3-positive cell line Jurkat and PD-L1-positive cell line MDA-MB-231 were used for the analysis of binding activity. 1×10^5^ cells were incubated with purified CiTE (CD3-BAFF-R-PD-L1) at 1.25 nM in a volume of 50 μL for 30 min. After washing three times with cold PBS, the cells were incubated with 100 μL Alexa Fluor 647 labeled anti-His tag antibody (MBL, Japan) at 0.5 g/mL for an additional 30 min. Then the stained cells were washed and analyzed by flow cytometry (LSRFortessa, Becton Dickinson Bioscience, San Jose, CA).

### Cytotoxicity and cell viability assays

To evaluate the effects of the tri-specific antibody on tumor cell growth, Raji cells were prepared as target cells. Human peripheral blood mononuclear cells (PBMCs) isolated from healthy volunteers served as effector cells. Different proportions of cells were seeded into 96-well round-bottom microplates and treated with the tri-specific antibody at different concentrations. The specific lysis of target cells was detected by CytoTox 96® Non-Radioactive Cytotoxicity Assay (G1780, Promega), according to the manufacturer's protocol. For the Chidamide-enhanced cytotoxicity assay, the optimal dose of Chidamide (1 μM) was added to the co-culture system. Raji cells were pretreated with or without Chidamide (1 μM), and 48 hours later, target cells were plated into 96-well plates (1×10^4^/well), with PBMCs added as indicated. The secretion of IL-2, TNF-α, and IFNγ in the supernatants was measured by ELISA kit (Neobioscience, Shenzhen).

### Expression of immune-related molecules in lymphoma cells after treatment with chidamide

2 × 10^5^ Z138, Jeko-1, or SU-DHL-10 cells were cultured in 24-well plates overnight. After treatment of Chidamide (1 μM) for 48 hours, total RNA was extracted using Trizol reagent (Invitrogen, USA). cDNA was synthesized from 2 μg of total RNA using Oligo(dT) primers and M-MLV reverse transcriptase (Invitrogen, USA). qPCR was performed on a QuantStudio 3 real-time PCR system (Applied Biosystems, USA) using SYBR Green (Takara, Dalian, China). The primers for individual genes are listed in Table 1. For flow cytometry analysis, lymphoma cells were collected and stained with antibodies against CD80, CD86, or Human leukocyte antigen antibodies to detect the expression levels.

## Results

### Design and production of CiTE (CD3-BAFF-R-PD-L1)

To construct CiTE (CD3-BAFF-R-PD-L1), Fab derived from a CD3 antibody was fused with the two identical scFvs targeting BAFF-R and PD-L1, as shown in Figure 1A. The fusion gene fragment was inserted into a pcDNA3.1(+) expression vector, which was expressed in adherent 293 T cells. A hexahistidine-tag (6×His-tag) was added to the N-terminus of the construct to facilitate detection and purification. Western blot results showed that the molecular weight of CiTE (CD3-BAFF-R-PD-L1) was approximately 110.55 kDa, while that of BiTE (CD3-BAFF-R) was about 57.01 kDa (Fig. 1B). Due to molecular interactions, a band at 114 kDa was observed, indicating that some of the BiTE products formed dimers.

### CiTE (CD3-BAFF-R-PD-L1) induces potent cytotoxic activity against BAFF-R positive target cells

**Figure 2:**
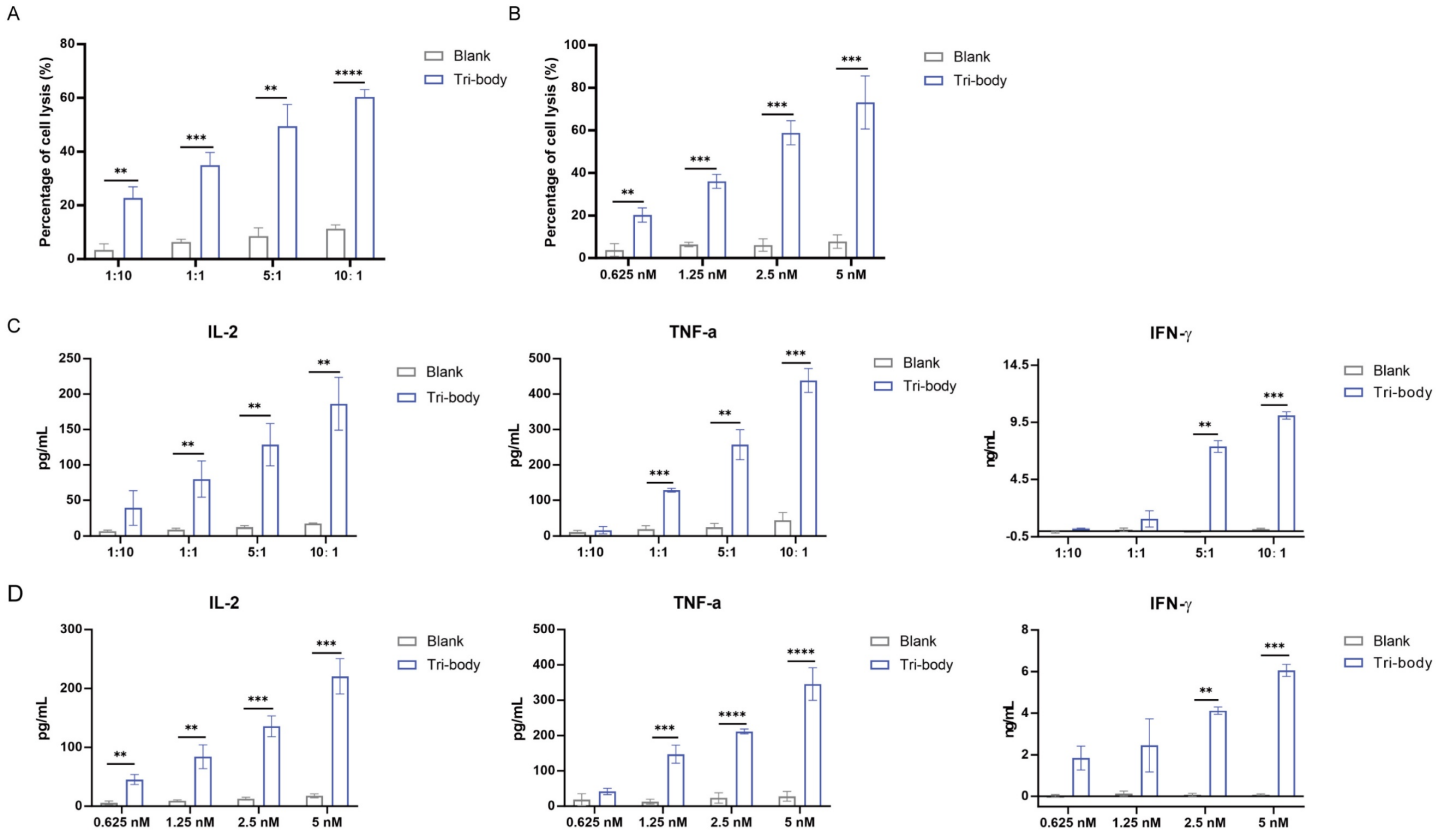
** The specific interaction between PBMCs and BAFF-R-positive cell lines induced by CiTE (CD3-BAFF-R-PD-L1). (A)** Specific lysis of target cell lines mediated by CiTE (CD3-BAFF-R-PD-L1). LDH release assays performed on Raji cells treated with CiTE (CD3-BAFF-R-PD-L1) (1.25 nM) in the presence of PBMCs in different effector to target (E:T) ratios for 24h. **(B)** Specific lysis of target cells induced by different concentrations of CiTE (CD3-BAFF-R-PD-L1) at the same E:T ratio (1:1) was also determined. **(C)** Cytokines including IL-2, IFN-γ, and TNF-α in the co-culture supernatants at different E:T ratio were detected by ELISA kits. **(D)** Cytokines including IL-2, IFN-γ, and TNF-α in the co-culture supernatants at E:T ratio (1:1) were detected by ELISA kits. *P < 0.05; **P < 0.01; ***P < 0.001 compared with the blank group. Data shown are the mean ± SD of three independent experiments.

**Figure 3:**
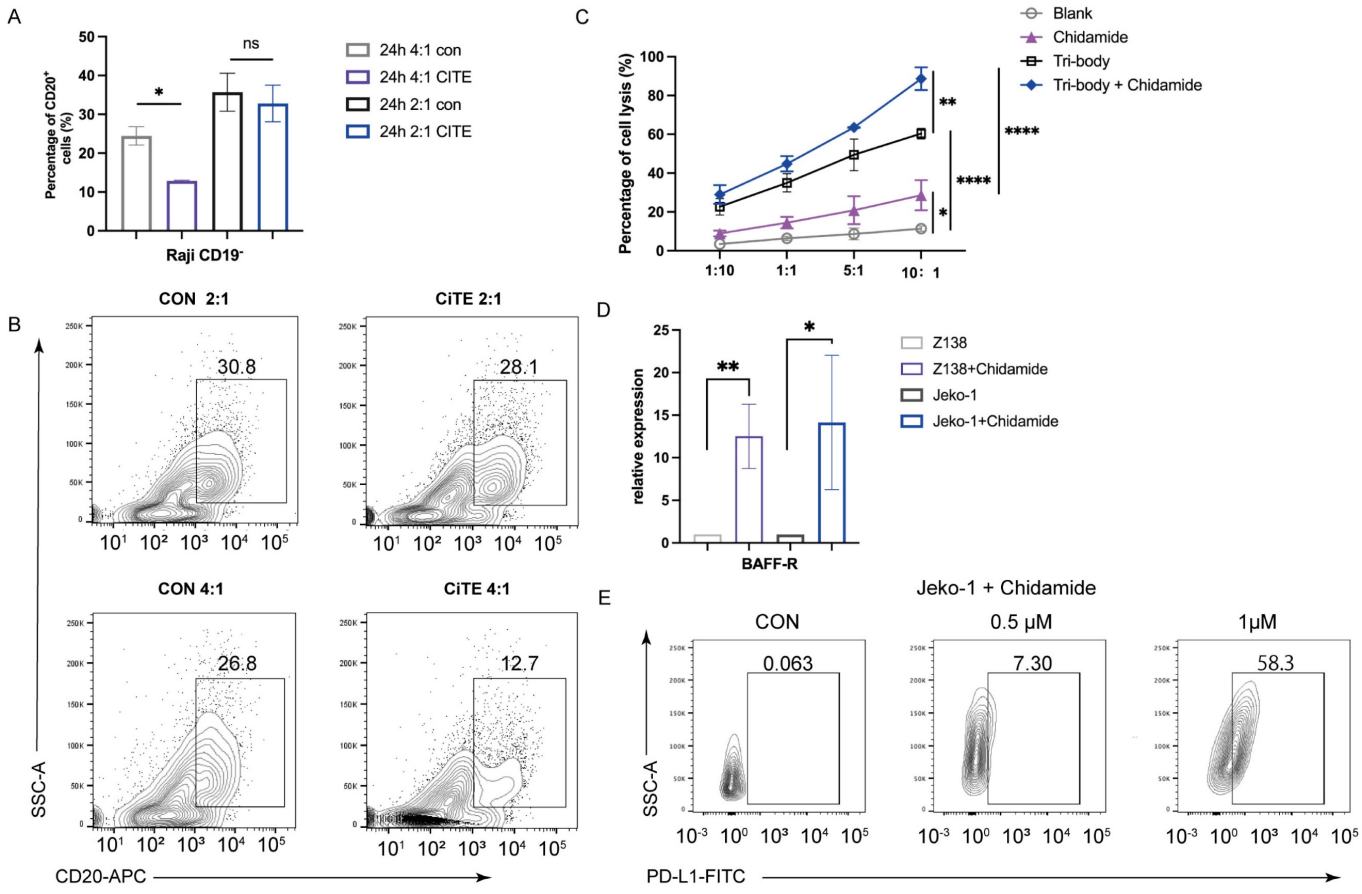
** Synergistic Antitumor Effects of CiTE and Chidamide in B-Cell Lymphoma Through Immune Modulation. (A, B)** The percentage of CD19^-^ Raji cells after being co-cultured with PBMC for the indicated time was determined by flow cytometry. **(C)** Cytotoxicity of PBMCs to Raji cells induced by CiTE (CD3-BAFF-R-PD-L1) with or without Chidamide (1μM). **(D)** Relative mRNA expression of BAFF-R in Z138 and Jeko-1 cells that were exposed to Chidamide (1 μM) for 48 h. **(E)** PD-L1 on Jeko-1 cells after treatment of Chidamide for 48 h. *P < 0.05; **P < 0.01 compared with the blank group. Data shown are the mean ± SD of three independent experiments.

The binding specificities of the purified CiTE (CD3-BAFF-R-PD-L1) were demonstrated by flow cytometry analysis on BAFF-R-positive Raji cells, CD3-positive Jurkat cells, and PD-L1-positive MDA-MB-231 cells (Fig. 1C). To determine the anti-tumor effect in the presence of activated human T cells, CytoTox96VR Non-Radioactive Cytotoxicity Assay was performed. BAFF-R-positive cells served as targets and were co-cultured with PBMCs at different E:T ratios ranging from 10:1 to 1:10. After treatment of CiTE (CD3-BAFF-R-PD-L1) (1.25 nM) for 24 hours, lysis was measured by lactate dehydrogenase release assay (Fig. 2A). Additionally, antibody-induced lysis was measured under the same E:T ratio (1:1) with different concentrations of CiTE (CD3-BAFF-R-PD-L1), from 0.625 nM to 5 nM (Fig. 2B). Tumor cell lysis was concentration-dependent, increasing with the E:T ratio or the concentration of CiTE (CD3-BAFF-R-PD-L1). Cytokines including IL-2, IFN-γ, and TNF-α were also measured to evaluate lymphocyte activation. Consistent with lysis results, cytokine levels also increased with the E:T ratio or the concentration of CiTE (CD3-BAFF-R-PD-L1) and were higher than those in the control groups (Fig. 2C-D). To confirm that CiTE (CD3-BAFF-R-PD-L1) induces lysis in CD19-negative cells, a CD19 KO Raji cell line was established. The percentage of CD20^+^ cells was detected at different E:T ratios, indicating that BAFF-R could serve as an alternative target in lymphoma treatment (Fig. 3A, B).

### Chidamide synergizes CiTE (CD3-BAFF-R-PD-L1)-mediated cytotoxicity

Epigenetic drugs have been shown to overcome the resistance of PD-1/PD-L1 therapy and to induce the production of tumor-associated antigens 19, 20. Chidamide is a potential treatment for B-NHL and has been reported to reverse CD20 deficiency caused by Rituximab immunotherapy 21, as well as synergize with anti-PD-L1 antibodies to reduce tumor burden by enhancing the immune function 22. We next evaluated whether Chidamide could synergize CiTE (CD3-BAFF-R-PD-L1)-mediated cytotoxicity. Raji cells were co-cultured with PBMC at different ratios following treatment with a low dose of Chidamide (1μM) for 48h. The CiTE (CD3-BAFF-R-PD-L1) group showed 60.37 ± 2.21%, 49.43 ± 6.65%, 34.99.73 ± 3.84%, and 22.70 ± 3.45% lysis at 10:1, 5:1, 1:1 and 1:10 ratio, respectively. After the pretreatment of Chidamide, lysis improved to 88.65 ± 4.79%, 63.51 ± 0.16%, 44.80 ± 3.23%, and 29.00 ± 3.93% (Fig. 3C). The combination index (CI) was 0.40 at 10:1 ratio, indicating that Chidamide synergized with CiTE-mediated T-cell killing.

To further explore the mechanism behind Chidamide-enhanced lysis, related antigens in Raji cells pretreated with Chidamide were measured. Chidamide increased BAFF-R expression in Raji cells (Fig. 3D), indicating enhanced binding probability for CiTE (CD3-BAFF-R-PD-L1). Additionally, tumor-associated antigens such as MAGEA1, MAGEA3, and GTAp63 were also induced by Chidamide, promoting antigen presentation and T-cell recognition (data not shown). These results indicate that Chidamide enhanced antibody binding and improve the cytotoxic effects of PBMCs.

### Antitumor potential of CiTE (CD3-BAFF-R-PD-L1) in combination with Chidamide against Raji xenograft tumors

**Figure 4:**
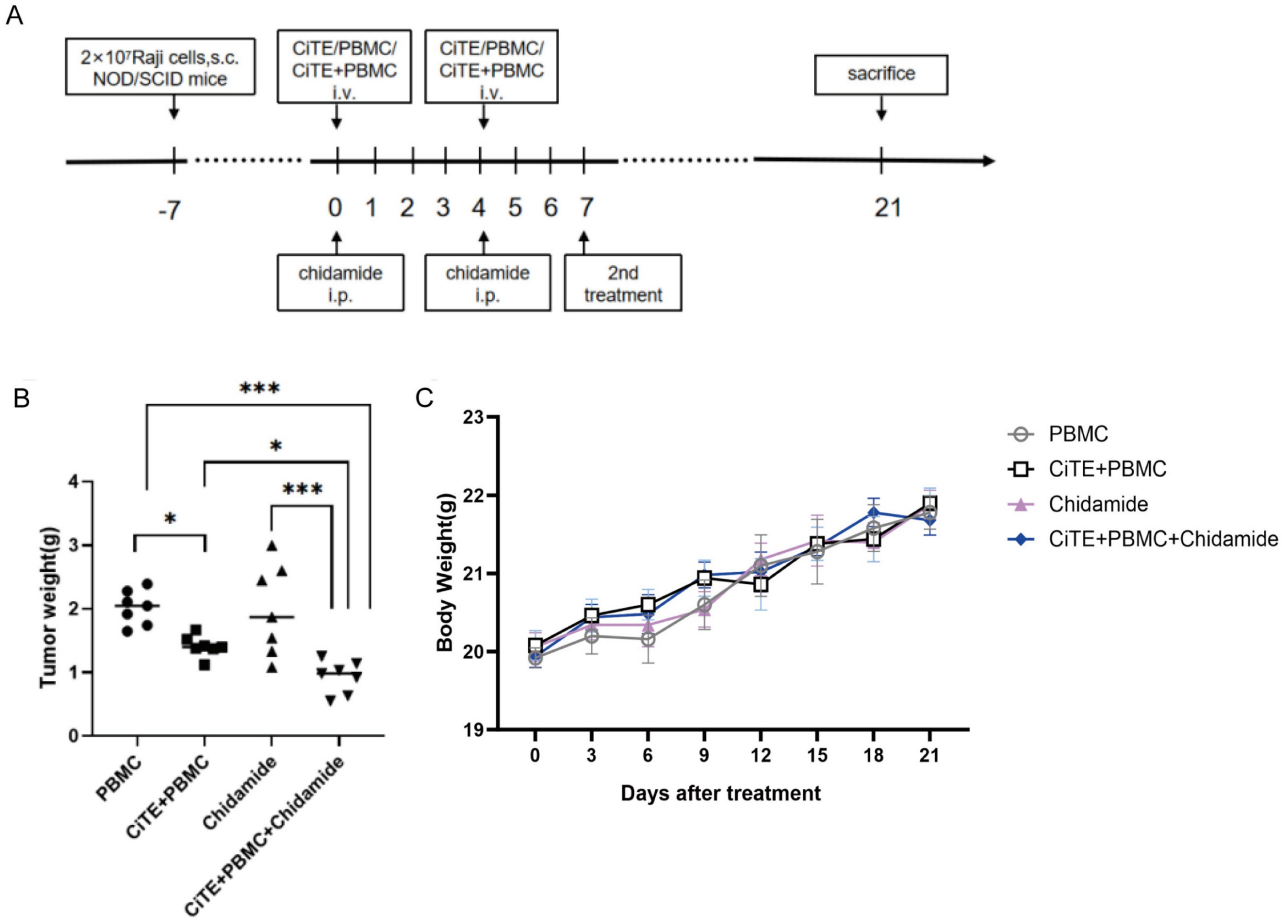
** Tumor suppressing effects of CiTE (CD3-BAFF-R-PD-L1) in combination with Chidamide against B cell lymphoma in mice. (A)** Experimental protocol of the tumor therapy. NOD/SCID mice were injected subcutaneously with Raji cells (2 × 10^7^ per mouse) into the right flank. Seven days later (day 0), Chidamide (3.9mg/kg) was administered to the xenograft Raji tumor-bearing mice via intraperitoneal injection. CiTE (CD3-BAFF-R-PD-L1) (1mg/kg) and / or PBMCs (5 × 10^6^ per mouse) were injected intravenously into the mice. The drugs were administered twice weekly. The second treatment was received on day 7. All mice were sacrificed on day 21. **(B)** The tumor weights of different groups were measured in the end of treatment. **(C)** Changes in the body weight of Raji lymphoma xenograft models during the treatment. *P<0.05; ***P<0.001

We investigated the antitumor potential of CiTE (CD3-BAFF-R-PD-L1) combined with Chidamide in BALB/c nude mice subcutaneously implanted with Raji cells. When the tumors reach a size of 100-200 mm^3^, mice were randomly divided into four groups (7 mice per group) and the experiment was conducted according to the planned protocol (Fig. 4A). The animals were sacrificed on day 21, and tumor tissues were dissected and weighed for analysis. Compared with PBMC control group, the mice treated with CiTE (CD3-BAFF-R-PD-L1) and CiTE (CD3-BAFF-R-PD-L1) + Chidamide showed significant tumor growth inhibition (Fig. 4B). In addition, no obvious changes in the body weight of the mice were observed during the treatment (Fig. 4C).

### Immunomodulatory effects of chidamide

**Figure 5:**
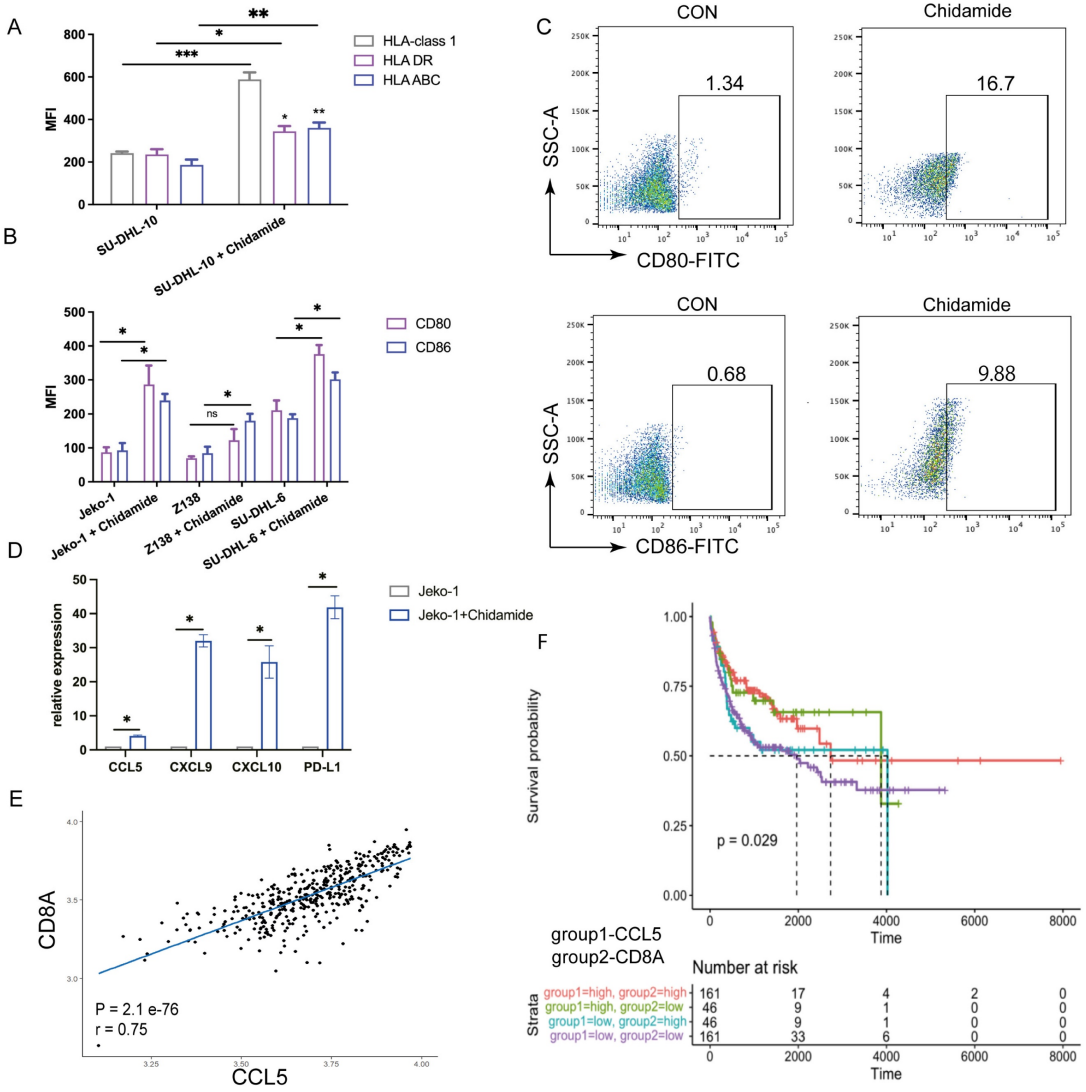
** Expression of costimulatory and human leukocyte antigen in B-NHL cell lines in the presence of Chidamide. (A)** Human leukocyte antigen (HLA-class1, HLA DR, HLA ABC) on SU-DHL-10 cells after treatment of Chidamide. **(B)** Expression of CD80 and CD86 on B-NHL cell lines in the presence of Chidamide. **(C)** Representative cytograms for the expression levels of CD80 and CD86 on Jeko-1 cells that were exposed to Chidamide for 48 h. **(D)** Relative mRNA expression of CCL5, CXCL9, CXCL10, and PD-L1 compared to vehicle (set to fold change = 1) in Jeko-1 cells that were exposed to Chidamide for 48 h. **(E)** Scatterplots showing the range of associations (r) with 95% CI and proportionality of expression levels for CD8A and CCL5 in DLBCL. **(F)** Kaplan-Meier survival curves in DLBCL as stratified by CCL5 and CD8A expression status using the GEO database. *P < 0.05; ****P < 0.0001 compared with the blank group. Data shown are the mean ± SD of three independent experiments.

To further explore the synergy between CiTE (CD3-BAFF-R-PD-L1) and Chidamide, we examined PD-L1 and tumor-associated antigens in B-NHL cell lines. Chidamide treatment upregulated PD-L1 expression on tumor cells in a dose-dependent manner over 48 hours, increasing tumor sensitivity to PD-1/PD-L1 antibodies (Fig. 3E). Consistent with previous studies 21, Chidamide induced the expression of costimulatory molecules (Fig. 5A) and Human Leukocyte Antigen (HLA-class I, HLA-DR, HLA-ABC). Co-stimulatory molecules CD80 and CD86 were also induced by Chidamide in B-NHL cell lines, enhancing CTL-mediated killing (Fig. 5B, C).

### Chidamide upregulates the expression of effector T cell-attracting chemokines

It is well known that the number of tumor-infiltrating lymphocytes is closely related to the efficacy of immunotherapy agents. Studies have shown that cytokines that recruit T cells are associated with clinical outcomes in cancer patients 23. qPCR on treated Jeko-1 cells revealed that the expression levels of CCL5, CXCL9, and CXCL10 significantly increased after Chidamide treatment, indicating that Chidamide enhances antibody function by increasing lymphocyte infiltration (Fig. 5D). Previous studies have also shown that high intratumoral expression of CCL5, a CD8+ T cell-associated chemokine, is associated with a better prognosis in various cancers 24. Analysis of the GEO database (GSE10846) revealed a positive correlation between CD8A and CCL5 expression (Fig. 5E), and CCL5^High^CD8A^High^ expression was associated with a lower risk of death in DLBCL (Fig. 5F). This indicates that Chidamide can act as an immunomodulator synergizing with CiTE (CD3-BAFF-R-PD-L1) against B-NHL.

## Discussion

In this study, we explored a novel combination of antibodies targeting CD3, BAFF-R, and PD-L1, and investigated the therapeutic potential of combining them with an HDAC inhibitor to enhance anti-tumor effects by modulating antigenic strength and the immunosuppressive environment.

Although bispecific antibody therapy is effective in treating B-cell malignancies, drug resistance due to target loss frequently occurs. Nearly 30% of relapses after CD19-directed BiTE or CAR T-cell therapy are due to CD19 deletion, underscoring the urgent need for alternative targets 25. The clinical application of T-cell therapy against BAFF-R is particularly promising for treating antigen loss in relapsed/refractory B-ALL following CD19-targeted therapy. While the BAFF/BAFF-R axis has been successfully utilized in autoimmune diseases, its efficacy in tumors remains unclear. Recently, monoclonal antibodies targeting BAFF-R have shown preclinical efficacy against CLL, especially when combined with BTK inhibitors 26. Additionally, several BAFF-R-targeted CAR-based studies are underway for the treatment of B-cell malignancies 27.

In this study, we constructed a CD3-BAFF-R-PD-L1 fusion protein, composed of anti-human CD3, BAFF-R, and PD-L1 scFv sequences. The structure mirrors that of Blinatumomab, a marketed bispecific antibody, with two hybrid scFv fragments fused into a single-chain polypeptide using a short linker peptide (Gly-Gly-Gly-Gly-Ser, G4S). VH and VL regions from each monoclonal antibody were separated by a longer (G4S)3 linker, and a 6×His-tag was added to the N-terminus for detection and purification of the fusion proteins.

We assessed the surface binding of the tribody using flow cytometry on various Non-Hodgkin lymphoma cell lines. Specifically, its binding ability to CD19-knockout Raji cells was tested, demonstrating its potential in managing CD19-negative relapsed B-cell malignancies. Further analysis of the CiTE (CD3-BAFF-R-PD-L1) function included tumor lysis assessment via the CytoTox96VR Non-Radioactive Cytotoxicity Assay and cytokine measurement using ELISA.

Epigenetics plays a fundamental role in cellular processes critical to cancer and immune cells, particularly in regulating T cell differentiation and function 28, which impacts antitumor immune responses 29. Exhausted T cells may contribute to anti-PD-1 treatment resistance by disrupting epigenetic homeostasis.

Several studies have highlighted the connection between epigenetic drugs and tumor immunogenic cell death, suggesting that epigenetic modulators can regulate the tumor microenvironment (TME) and the immune cycle by enhancing the expression of tumor-associated antigens, MHC molecules, and antigen-presenting cells (APCs), thus promoting immune cell priming and tumor antigen recognition 30. Epigenetic drugs have also shown therapeutic synergy when combined with immune checkpoint blockade drugs, leading to increased effector T-cell chemokine production and activation of effector T cells. Various histone modifications, such as acetylation, methylation, phosphorylation, ubiquitination, ADP-ribosylation, SUMOylation, and citrullination, regulate the TME and immune cells 30.

HDACs are a large family of proteins categorized into five classes: class I (HDAC 1, 2, 3, 8), class IIa (HDAC 4, 5, 7, 9), class IIb (HDAC 6, 10), class III (Sirtuins), and class IV (HDAC 11). Chidamide, a selective inhibitor targeting HDAC1, HDAC2, HDAC3, and HDAC10, has been approved for treating relapsed/refractory peripheral T cell lymphoma and hormone receptor-positive, HER2-negative breast cancer after endocrine therapy 31. Several clinical trials are underway exploring its use in B-NHL treatment.

Chidamide has been reported to modulate the TME and overcome anti-PD-1 therapy resistance 20. In this study, we demonstrated that Chidamide upregulates PD-L1 and BAFF-R in NHL, enhancing the binding affinity of CiTE (CD3-BAFF-R-PD-L1). The elevated PD-L1 induced by Chidamide was effectively blocked by the CiTE construct while serving as a binding target for the tribody, thereby strengthening the antitumor effect. This reversed the immunosuppressive tumor phenotype to an immune-activating state.

Additionally, we demonstrated that Chidamide promotes the secretion of several chemokines that attract CD8+ T cells in NHL, including CXCL9, CXCL10, and CCL5. The loss of tumor-intrinsic chemokines supporting T-cell recruitment is a common mechanism of immune evasion and resistance to immune checkpoint blockade. We highlighted Chidamide's role in inducing CCL5 expression, which is often suppressed by epigenetic silencing mechanisms 24. Consistent with findings in solid tumors, CD8+ T cell infiltration is strongly associated with CCL5 in DLBCL, and our analysis of GEO datasets revealed that high intratumoral CCL5 expression correlates with better prognosis and increased intratumoral CD8A expression in DLBCL. We hypothesize that Chidamide alters the TME, creating an immune-activating environment that enhances anti-PD-1 therapy by recruiting more lymphocytes.

Moreover, we confirmed the upregulation of HLA class I and II molecules and the induced expression of co-stimulatory molecules 32, in line with the known positive correlation between MHC class II expression and PD-1/PD-L1 response 33. We speculate that Chidamide sensitizes tumors to the tribody's antitumor effect by regulating antigen presentation processes. Combined with chemokine upregulation, Chidamide transforms the immunosuppressive microenvironment and promotes neoantigen production, acting as an immunomodulator that works synergistically with antibodies to eliminate tumors.

However, the efficacy of the CD3-BAFFR-PD-L1 tribody may vary among different B-cell lymphoma subtypes due to differences in BAFFR and PD-L1 expression levels. Additionally, tumor microenvironment heterogeneity, including variations in immune cell composition and cytokine profiles, may impact drug distribution, T-cell activation, and therapeutic response 34. Previous studies have reported that HDAC inhibitors can modulate the tumor microenvironment by upregulating chemokines such as CXCL10 and CXCL12, thereby enhancing immune cell infiltration 35,36. However, since our study was conducted in immunodeficient mice, we were unable to fully assess the immune regulatory effects of Chidamide *in vivo*. Future studies should explore these aspects in immunocompetent models and clinical samples to better understand the impact of tumor heterogeneity on treatment outcomes. We have incorporated this discussion into the revised manuscript to provide a more comprehensive perspective on our findings.

## Conclusions

In conclusion, we established CiTE (CD3-BAFF-R-PD-L1) as an alternative therapeutic target for lymphoma. The combination of Chidamide and tribody may synergistically reduce tumor burden by enhancing the immune response. This discovery provides a compelling rationale for investigating this combination therapy in clinical trials to overcome immunosuppression and improve patient outcomes in malignancies.

## Figures and Tables

**Table 1:** List of primers

Primer Names	Primer Sequence (5'-3')
CCL5 F	CGCTGTCATCCTCATTGCTA
CCL5 R	GCACTTGCCACTGGTGTAGA
CXCL9 F	GGCATCATCTTGCTGGTTCT
CXCL9 R	TCACTCTGCTGAATCTGG
CXCL10 F	CTTTCTGACTCTAAGTGGCATTC
CXCL10 R	CACCCTTCTTTTTCATTGTAGCAA
BAFF-R F	GGAAGACCCAGGAACCAC
BAFF-R R	TTTGGTGTGCTTGCCTT
CD86 F	TGATTCGGACAGTTGGACCCTGAGAC
CD86 R	AAGGTGAAGATAAAAGCCGC
CD80 F	GTCCAAATTGTTGGCTTTCA
CD80 R	GAAGAATGCCTCATGATCCC
MAGEA1 F	GCCTGCTGCCCTGACGAGAG
MAGEA1 R	AGGAGAGACCTAGGCAGGTG
MAGEA3 F	ATTCTCGCCCTGAGCAACGAG
MAGEA3 R	GACGACACTCCCCAGCATTT
GTAp63 F	ATTCCGGACACCCTATCAGAG
GTAp63 R	CCCAGATATGCTGGAAAACCT
